# Iatrogénie médicamenteuse chez la personne âgée: à propos de deux cas d’insuffisance rénale favorisée par les tricycliques

**DOI:** 10.11604/pamj.2018.30.282.14947

**Published:** 2018-08-23

**Authors:** Sodjehoun Apeti, Mawufemo Yawovi Tsevi, Matchonna Kpatcha, Eyram Yoan Amekoudi, Akomola Kossi Sabi

**Affiliations:** 1 Service de Médecine Interne, CHU Sylvanus Olympio, Université de Lomé, Togo; 2 Service de Néphrologie et d’Hémodialyse, CHU Sylvanus Olympio, Université de Lomé, Togo; 3 Service d’Urologie, CHU Sylvanus Olympio, Université de Lomé, Togo

**Keywords:** Iatrogénie, personnes âgées, tricycliques, hypertrophie bénigne prostatique, insuffisance rénale, Iatrogenia, elderly, tricyclics, benign prostatic hyperplasia, renal failure

## Abstract

L'hyperplasie prostatique est une affection courante chez l'homme à partir de la cinquantaine dont l'incidence augmente avec l'âge. Elle est préoccupante lorsqu'elle est symptomatique. Les médicaments ayant des effets néfastes sur le fonctionnement du bas appareil urinaire, peuvent favoriser l'apparition des symptômes. Parmi ceux-ci, les médicaments à effets primaires ou secondaires anticholinergiques dont les tricycliques sont souvent en cause. Ils sont contre-indiqués en cas de pathologie obstructive sous vésicale parce que pouvant être responsable de l'apparition ou l'aggravation des symptômes urinaires et des complications qui en découlent. La plus grave est l'insuffisance rénale avec ses conséquences sanitaires et économiques chez les sujets âgés souvent polypathologiques. Nous rapportons deux cas de iatrogénie des tricycliques sur l'appareil urinaire chez des patients âgés de 80 et 92ans porteurs d'hyperplasie prostatique. L'imagerie et le Bladder scan ont permis de poser le diagnostic étiologique du fait de la pauvreté sémiologique permettant une prise en charge adéquate. Ces observations justifient la réalisation systématique du toucher rectal, du Bladder scan voire d'une échographie chez tout sujet âgé présentant une insuffisance rénale aiguë ou une aggravation d'une insuffisance chronique du fait de la fréquence des pathologies prostatiques et de la polymédication à cet âge.

## Abstract (en)

Prostatic hyperplasia is a common condition in people over the age of 50 and its incidence increases with age. It gives particular cause for concern when it is symptomatic. Drugs with potential adverse effects on lower urinary tract can induce symptoms. Among those, anticholinergic drugs causing primary or secondary effects, such as tricyclics, are often associated with symptoms. These are contraindicated in patients with subvesical obstruction because they can induce or worsen urinary symptoms and complications resulting from them. Renal failure having health and economic consequences on elderly subjects Who often suffer from multiple disorders is the most severe complication. We report two cases of tricyclics-related adverse effects on the urinary tract in patients with prostatic hyperplasia ranging between 80 and 92 years. Given the lack of semiologic signs, imaging techniques and bladder TC scan enabled etiological diagnosis allowing adequate management. Study results justify a systematic performace of rectal examination, bladder CT scan or even ultrasound in any elderly with acute renal failure or with a worsening of chronic renal failure based on the frequency of prostatic pathologies and polypharmacy in this age.

## Introduction

L'iatrogénie médicamenteuse est un événement fréquent en pratique médicale chez des patients hospitalisés ou en ambulatoire [1-3]. La prescription médicamenteuse chez le sujet âgé s'appuie sur cet aspect en tenant compte des effets indésirables graves mais aussi des précautions d'emploi du fait des modifications pharmacologiques du médicament lié au vieillissement des organes [4-7]. Ainsi, il est recommandé de réviser régulièrement les ordonnances du sujet âgé pour une prescription appropriée [4,8]. En effet parmi les médicaments les plus consommés par la population gériatrique, les psychotropes y occupent une place importante dont font partie les antidépresseurs [7,9,10]. De surcroit, beaucoup sont sous les antidépresseurs classiques souvent tricycliques avec leur effet anticholinergique sur les muscles lisses des organes comme la vessie, l'œil, l'intestin et sur le système nerveux central aux conséquences graves [6,11]. Il est à noter que ces antidépresseurs peuvent être prescrits avant l'entrée dans l'âge gériatrique. Les antidépresseurs tricycliques sont recommandés en 2^ème^ ou 3^ème^ intention de même que les IMAO sélectifs ou non [7]. L'hypertrophie prostatique symptomatique ou non peut être une cause d'insuffisance rénale obstructive et constitue une contre-indication absolue des tricycliques [6,8,11]. Par ailleurs, il est recommandé qu'une cause médicamenteuse soit systématiquement évoquée devant tout nouveau symptôme et toute altération de l'état de santé d'une personne âgée si l'explication n'est pas d'emblée évidente [5,11]. Nous rapportons dans cette étude deux cas cliniques ayant retenu notre attention. Ce travail a pour objectif général de rappeler le réflexe iatrogénique des médicaments chez les prescripteurs gériatriques dans la prise en charge des personnes âgées et spécifiquement d'exposer les graves complications des tricycliques au niveau de l'appareil urinaire.

## Patient et observation

**Observation 1:** Un homme de 80ans a été hospitalisé dans le service de médecine gériatrique pour altération de l'état général évoluant depuis plus d'un an avec asthénie, anorexie, amaigrissement de 7kg environ en 6mois (55 kg contre 62 kg il y a 6 mois), nausées et constipation intermittente. La persistance de la constipation depuis plus d'une semaine a motivé son admission pour prise en charge. Il n'y avait pas de symptômes du bas appareil urinaire (pollakiurie, dysurie, nycturie, urgenturie, sensation de vidange incomplète, diminution de la force du jet, flux intermittent). Le bilan biologique aux urgences avait retrouvé une insuffisance rénale avec une clairance rénale terminale (MDRD = 8ml/mn/1,73m^2^). La Radiographie de l'abdomen sans préparation était sans particularité en dehors d'une stase stercorale sans fécalome. Dans les antécédents, on notait une dépression de plus de 20ans sous Clomipramine (Anafranil) 75mg par jour, une notion de zona intercostal sans cicatrice séquellaire ayant justifié en plus l'Anafranil pour prévenir les douleurs postzostérennes selon son médecin traitant. Il est autonome sur le plan physique et cognitif. Il est l'aidant naturel de sa femme en rééducation fonctionnelle pour un AVC depuis environ 6 mois dans un service de soins de suite et de réadaptation. Ses plaintes actuelles sont rattachées à l'état de santé de sa femme par son entourage et son médecin traitant. Le seul bilan rénal retrouvé chez son médecin traitant remonte à Avril 2013 montrant un début d'insuffisance rénale avec un MDRD à 58ml/mn/1,73m^2^. Son traitement personnel est constitué de Clomipramine (Anafranil) 75mg/j et de Dompéridone 10 mg x3/j mise il y a deux mois pour les nausées. L'examen clinique était sans particularité en dehors d'une prostate un peu volumineuse, ferme et indolore au toucher rectal (TR). La diurèse était de 2litres avec un apport hydrique de 1,5l/24heures aux sorties des urgences. L'évaluation gériatrique standardisée était normale en dehors d'un score MNA à 22 avec un IMC à 19,5kg/m^2^ (18,4kg/m^2^ après sondage et évacuation de 4400ml d'urine en 24heures).

**Le bilan paraclinique** A l'entée.

**Sur le plan biologique:** A la première heure, urée à 31,7mmol/l et créatininémie à 608μmol/l (MDRD à 8ml/mn/1,73m^3^), hyperkaliémie à 4,9mmol/l, syndrome inflammatoire biologique avec CRP à 141mg/l et polynucléose neutrophile (PNN) à 10330/mm^3^ (normes: 1800 et 6980PNN/mm^3^), lymphopéne à 910/mm^3^(normes: 1260 et 3350/mm^3^), hyperfibrinogénémie à 7,04g/l (normes: 2,38 et 4,98 g/l). Après 24 heures, urée à 29,7mmol/l et créatininémie à 633μmol/l (MDRD à 8ml/mn/1,73m^3^), hyperkaliémie à 4,8mmol/l, hyperLDHémie à 311 UI/l, hypocalcémie à 2,04mmol/l, albuminémie corrigée (selon CRP) normale à 36,4 g/l, PNN à 8130/mm^3^.

**Sur le plan radiographique:** L'ASP aux urgences note une stase stercorale sans fécalome. Devant ce tableau, nous avons arrêté le traitement antidépresseur tricyclique (Anafranil) et la Dompéridone et demandé un bilan pour explorer le syndrome inflammatoire (ECBU, Electrophorèse des protides, Marqueurs tumoraux), pour contrôler le fonction rénale et un scanner thoraco-abdomino-pelvien du fait du syndrome inflammatoire dont les résultats sont à J1 dans le service.

**En biologie:** MDRD à 8ml/mn/1,73m^2^ (créatininémie à 656 μmol/l), hypoalbuminémie non corrigée à 29,9g/l, hyperalpha-1-globuline à 4,48gg/l et hyperalpha-2-globuline à 14,6g/l, gammaglobuline normale faisant évoquer un syndrome inflammatoire modéré avec hypoalbuminémie. Les marqueurs tumoraux dont le PSA étaient normaux en dehors de CA 15-3 qui était augmentée à 45 UI/ml. Le reste du bilan gériatrique standard était normal et l'ECBU était négatif.

**En imagerie:** Le scanner thoraco-abdomino-pelvien avait retrouvé des reins de taille normale, une dilatation pyélique bilatérale avec un bassinet mesurant à droite 25mm et à gauche 20mm, une hypotonie urétérale droite. Il mettait en évidence un calcul de 6mm de diamètre enclavé au niveau du méat urétéral droit. Il n'y avait pas d'obstacle à gauche en dehors d'un syndrome de jonction. La vessie était pleine à paroi régulière. On note une hypertrophie prostatique avec un gros lobe médian. Le diagnostic de rétention urinaire sur hypertrophie prostatique avec lithiase urétérale droite responsable d'insuffisance rénale obstructive avec urétérohydronéphrose bilatérale a été retenu. Un sondage vésical a été effectué ramenant une diurèse de 4400ml/24heures. Un avis urologique et néphrologique avait été demandé pour un éventuel transfert mais il a été recommandé de garder la sonde et de surveiller le bilan rénal pendant 24 heures avant d'envisager un transfert en absence d'amélioration. L'évolution du tableau clinique et paraclinique a été favorable dès la 12^ème^heure du sondage. L'anorexie, l'asthénie et la constipation ont commencé par régresser avec reprise de l'alimentation. La clairance s'est améliorée progressivement avec un MDRD à J1 de 17ml/mn/1,73m^2^ (Urée à 17,7mmol/l et créatininémie à 334μmol/l), J2 de 36ml/mn/1,73m^2^ (Urée à 11mmol/l et créatininémie à 160μmol/l), J3 de 50ml/mn/1,73 m^2^ (Urée à 9,8mmol/l et créatininémie à 127μmol/l) et à J4 > 60ml/mn/1,73m^2^ (Urée 6,3mmol/l et créatininémie 72μmol/l). La kaliémie aussi s'est normalisée. A la consultation urologique, il a été décidé une résection du lobe médian par voie endoscopique. Mais au terme de l'intervention, tant le lobe médian, les lobes latéraux et apicaux avaient été réséqués de façon systématique sans raison particulière et la pièce opératoire envoyée en anatomopathologie. Les suites opératoires sont simples.

**Observation 2:** Un homme de 92ans a été hospitalisé dans le service de médecine gériatrique pour grabatisation évoluant depuis 48 heures avec asthénie, anorexie et constipation. Cette constipation auparavant intermittente était devenue persistante depuis une dizaine de jours. Il a été adressé aux urgences par son médecin traitant pour prise en charge. L'interrogatoire ne retrouve pas de symptômes du bas appareil urinaire. Le bilan aux urgences avait retrouvé une insuffisance rénale terminale (MDRD à 11 ml/mn/1,73m^2^). La Radiographie de l'abdomen sans préparation avait retrouvé une stase stercorale avec fécalome. Il est adressé en hospitalisation pour prise en charge de la constipation et réautonomisation. Il se déplace à domicile et vit à l'étage sans difficulté. Dans les antécédents on retrouve une tumeur cutanée du dos de 10cm x 8cm récusée depuis 5ans à cause de l'âge selon ses enfants mais en effet le patient n'aurait jamais consulté pour cela dans un hôpital conventionné lorsque nous avons recoupé les informations, une arythmie cardiaque par fibrillation auriculaire (AC/FA) ayant nécessité la pose d'un pacemaker avec des extrasystoles ventriculaires, une cyphose dorsale physiologique lié à l'âge et une hyperplasie prostatique sous Alfusozine 10mg par jour et Serenoa repens (Permixon) 160mg matin et soir, notion d'insuffisance rénale en 2014 et en 2016 avec MDRD respectifs à 39 et 46ml/mn/1,73m^2^. Son traitement personnel est constitué d'Amitryptiline (Laroxyl) 25mg matin et soir depuis plus de 20ans et Tianeptine (Stablon) 12,5mg trois fois par jour dont nous avons cherché l'indication justifiée en vain, de Piribédil (Trivastal) Lp 50 mg/j pour les tremblements, de Bisoprolol 2,5mg/j pour son AC/FA, de Formotérol (Foradyl) 01 bouffée matin et soir, et d'Aminophylline injectable (Minofil) 1 flacon matin sans aucune indication pulmonaire retrouvée, de Furosémide 40 mg matin pour les OMI et non pour l'insuffisance cardiaque , Paracétamol 1gx3/j pour les douleurs éventuelles, d'Alprazolam 0,25 mg (1/2comprimé) le soir pour insomnie, Salicylate de Lysine 75mg/jour pour prévention thromboembolique de son arythmie cardiaque, d'Alfusozine 10mg par jour et de Serenoa repens (Permixon) 160 mg matin et soir pour l'hyperplasie prostatique, de Febuxostat (ADENURIC) 80mg matin pour traitement de la goutte, de Renutryl boisson dans le cadre d'une dénutrition. L'examen clinique était sans particularité en dehors d'une hypotension artérielle à 80/40mmHg et d'une prostate augmentée de taille, ferme et indolore au toucher rectal (TR) et d'une lésion maligne du dos sous pansement palliatif. Un bladder scan systématique a retrouvé un contenu vésical estimé à plus de 999ml ayant justifié un sondage vésical. L'évaluation gériatrique standardisée était normale en dehors d'un score IADL à 2/4. Le MNA était à 26 avec un IMC à 29,4kg/m^2^. Le score GIR était à 3 à J0 puis à 5 à J2.

**Le bilan paraclinique** A l'entée.

**Sur le plan biologique:** Hyperglycémie à 1,27g/l, Urémie à 38,8mmol/l et créatininémie à 449μmol/l ( MDRD de 11ml/mn/1,73m^3^), hyperkaliémie à 5,9mmol/l et hypochlorémie à 96mmol/l, hyperLDHémie à 311UI/l, hyperlipasémie à 135UI/L, un syndrome inflammatoire biologique avec CRP à 179 mg/l et polynucléose neutrophile à 11800/mm^3^ (normes: 1800 à 6980 PNN/mm^3^), hyperfibrinogénémie à 5,15g/l (normes: 2,38 et 4,98g/l), lymphopéne à 910/mm^3^ (normes: 1260 et 3350mm^3^).

**Sur le plan radiographique:** L'ASP notait une stase stercorale avec fécalome et une cardiomégalie avec ICT à 0,62. Devant ce tableau, un ajustement thérapeutique a été effectué avec arrêt de Laroxyl, Stablon, Paracétamol, Alfuzosine, Furosémide, Trivastal, ADENURIC, Foradil, Minofil. Nous avons réduit la posologie du Bisoprolol à 1,25mg/jour, administré du sulfonate de polystyrène sodique (Kayexalate) en dose unique, de la Calciparine dose préventive, demandé un bilan d'exploration du syndrome inflammatoire, un bilan rénal contrôle et un scanner thoraco-abdomino-pelvien en plus de l'échographie abdomino-pelvienne. Nous avons demandé également une consultation cardiologique, urologique, pneumologique et dermatologique pour ajustement thérapeutique. Evolution et suivi dans le service: Devant cette rétention urinaire, nous avons posé une sonde vésicale ramenant une diurèse de 2500ml/24heures, instauré une prise en charge de la constipation avec laxatif oral et lavement.

**Sur le plan biologique:** A J1, MDRD à 15ml/mn/1,73m^2^ (créatininémie à 353 μmol/l et hyperurémie à 37,6mmol/l), hypoalbuminémie corrigée à 35,1g/l, hypobicarbonatémie à 19mmol/l, hyperphosphorémie à 1,65mmol/l, hypouricémie à 136mmol/l, proBNP élevée à 5205pg/ml, hypovitaminose D à 13,7μg/l. Le reste du bilan gériatrique notamment la calcémie, les hormones thyroïdiennes, l'hémostase était normal. A J2, MDRD à 26ml/mn/1,73m^2^ (créatininémie à 217μmol/l et hyperurémie à 33,8mmol/l). Le taux de PSA était à 38,66ng/ml, l'ECBU était négatif et la kaliémie normale. A J7, MDRD à 58ml/mn/1,73m^2^ (créatininémie à 109μmol/l et hyperurémie à 12,05mmol/l), hyperkaliémie à 4,9mmol/l. La Protidémie était à 59g/l (Normes: 60-80g/l). Nous avons tenté à cette date une ablation de la sonde mais le patient a eu une rétention aiguë d'urine d'où la nécessité d'une sonde à demeure.

**Sur le plan de l'imagerie:** L'échographie a montré une hypertrophie prostatique de poids estimée entre 70 et 75g, aux contours réguliers, un kyste polaire supérieur du rein droit de 72mm et gauche de 60mm. Une vessie anéchogène sans évaluation du résidu post-mictionnel. Le scanner abdominopelvien a retrouvé des reins de taille normale, bosselés, multikystiques bilatéraux, des kystes hépatiques épars et une stase stercorale colorectale avec fécalome, une disco-lombarthrose étagée. Il n'y avait pas de dilatation pyélique bilatérale avec un bassinet mesurant à droite 25mm et à gauche 20mm. On notait une hypotonie urétérale des cavités pyélocalicielles. La vessie était de plage homogène à paroi régulière. On notait une hypertrophie prostatique avec un gros lobe médian. A la consultation cardiologique à J7, l'ECG montre un rythme électro-stimulé en mode double chambre à 60bpm avec activité en flutter auriculaire associée. L'échographie cardiaque montre une oreillette gauche dilatée avec cinétique ventriculaire en fonction des troubles du rythme. Il a été mis sous Apixaban (Eliquis) 2,5mg matin et soir avec arrêt du salicylate de lysine. La consultation dermatologique a été refusée tant par le patient que son entourage, il est alors étiqueté pour pansements palliatifs. L'avis urologique a préconisé un rabotage du fait de l'échec de la tentative d'ablation de la sonde urinaire. Le patient reste encore non opéré pour des raisons d'indisponibilité lié au calendrier de l'urologue. La récupération de la marche était effective dès J1 avec l'aide de la kinésithérapie.

## Discussion

L'hyperplasie prostatique est une pathologie fréquente chez l'homme et représente une des 10 pathologies communes de l'homme à partir de la cinquantaine. Elle touche la moitié des hommes à partir de 60ans et va jusqu'à 90% chez les plus 80ans [12-14]. La rétention urinaire et les autres signes du bas appareil urinaire liés à l'hypertrophie prostatique sont les raisons de mise sous traitement dont la fréquence augmente avec l'âge. Cette fréquence est de 25% entre 50-59ans, 33% entre 60-69ans et 50% au-delà de 80ans [15]. Tout médicament pouvant favoriser l'aggravation des symptômes et conséquences des pathologies prostatiques nécessite une attention particulière. Beaucoup de médicaments à effet anticholinergique comme les tricycliques font partie de cette classe thérapeutique et favorisent la rétention urinaire avec ses conséquences néfastes [8,16,17]. Ces effets indésirables sont graves du fait des hospitalisations et de l'insuffisance rénale qui en découlent [8,16,17]. En effet une prescription non rationnelle chez le sujet âgé est source d'hospitalisation inutile [11,18,19]. Une étude du centre de pharmacovigilance en France a retrouvé que plus de 10% (5 à 10% à partir de 65 ans jusqu'à 20% à partir de 80ans) des hospitalisations sont iatrogéniques [5,11], dont les deux grandes classes thérapeutiques sont les psychotropes et les médicaments cardiovasculaires [20]. Ainsi selon l'étude STOPP-START les tricycliques doivent être proscrits ou arrêtés chez les personnes âgées pour leurs divers effets anticholinergiques sur le système nerveux, l'intestin, l'œil et surtout sur l'appareil urinaire d'autant plus que la pathologie prostatique est très fréquente chez les sujets âgés [8].

En effet, la iatrogénie est une double perte pour le financement de la santé car il est incohérent de dépenser pour acheter les médicaments et gérer les conséquences iatrogéniques des mêmes médicaments [5]. Nos deux patients ont présenté des tableaux caractéristiques de la iatrogénie aux tricycliques justifiant une proscription de ces molécules chez les sujets âgés du fait de l'existence d'alternative efficace comme les inhibiteurs de la recapture de la sérotonine [3,5,8]. Ils n'ont pas présenté des tableaux de signes de bas appareil urinaire, ce qui constituent des difficultés diagnostiques et pourraient justifier des égarements diagnostiques. Le patient serait adressé de spécialité en spécialité en absence d'analyse soigneuse de ses prescriptions médicamenteuse. La révision des ordonnances est une attitude qui doit faire partie de la démarche quotidienne des intervenants en milieu gériatrique tant dans le secteur public et privé [5,6,8]. Une hospitalisation est toujours une occasion de dégradation de l'état physique, psychique et social du sujet âgé et doit donc être évitée par de bonnes pratiques professionnelles [1,21-23]. A cet effet, la réalisation systématique d'un bladder scan devant tout sujet âgé surtout sous médicament à effet anticholinergiques permettra de dépister cette affection souvent cliniquement asymptomatique. La rétention chronique n'est pas aussi bruyante comme celle aiguë qui, est une cause classique de confusion chez le sujet âgé et alerte le praticien [5,23]. La récupération sous sondage n'a suivi que l'évolution normale des insuffisances rénales sur obstacles caractérisée par le syndrome de « levée d'obstacle (post obstructive diuresis des Anglo-Saxons) ». Sur le plan biologique les deux patients ont présenté une hyperkaliémie, une hyperLDHémie et un syndrome inflammatoire biologique avec élévation de la CRP, l'hyperfibrinogénémie et une polynucléose neutrophile progressivement normalisées avec l'amélioration de l'insuffisance rénale. Nos recherches dans la littérature médicale ne nous ont pas permis de trouver des arguments pouvant expliquer l'association de ces anomalies biologiques. La production des cytokines pro-inflammatoires dans l'insuffisance rénale pourrait jouer un rôle [6]. Toutefois, l'insuffisance rénale s'accompagne parfois d'une hyperkaliémie mais existerait-il un lien avec les tricycliques? Des études de grande envergure méritent d'être menées pour élucider ces constats. Le patient de 92ans a présenté une hypotension qui pourrait être l'action conjuguée du Furosémide, de l'Alfuzosine, du Bisoprolol et de l'Amitryptyline [1-3]. Enfin, le contexte social des deux patients serait faussement pris comme facteur de dégradation de leur état de santé alors qu'il y a une véritable pathologie organique en cause. Ainsi, la recherche d'affection organique doit être la hantise du médecin devant toute modification de l'état sanitaire du sujet âgé avant de le rattacher au contexte social.

## Conclusion

L'iatrogénie grave est une réalité fréquente chez la personne âgée et doit être prévenue par tous les moyens possibles. L'âge précoce de survenue des pathologies prostatiques impose une attention particulière sur les prescriptions d'avant et après 50ans. Une révision systématique des ordonnances dès l'âge de 50ans en général et à partir de 65ans en particulier à la recherche de médicament anticholinergique associé à un toucher rectal simple et au besoin à l'imagerie peuvent permettre d'éviter cette iatrogénie par maladresse des professionnels de santé. L'utilisation systématique de bladder scan peut être contributif. Par ailleurs, la fréquence élevée des maladies cardiovasculaire à cet âge impose les mêmes attitudes pour évaluer l'usage des alpha-bloquants dans le traitement de l'hypertrophie prostatique afin d'éviter l'hypotension en association avec les antihypertenseurs et autres vasodilatateurs.

## Conflits d’intérêts

Les auteurs ne déclarent aucun conflit d'intérêts.
